# Is waist to height ratio better at assessing cause-specific mortality risk than body mass index or waist circumference? A prospective analysis in a large U.S.-based cohort

**DOI:** 10.1371/journal.pone.0328760

**Published:** 2025-08-13

**Authors:** Alpa V. Patel, Kierstin Faw, Erika Rees-Punia, Brad Heltemes, Clara Bodelon, Anita Peoples, Lauren E. McCullough, Lauren R. Teras

**Affiliations:** 1 Department of Population Science, American Cancer Society, Atlanta, Georgia, United States of America; 2 Munich Re Life United States of America, New York, New York, United States of America; 3 Department of Epidemiology, Rollins School of Public Health, Emory University, Atlanta, Georgia, United States of America; Instituto Nacional de Cardiologia Ignacio Chavez, MEXICO

## Abstract

**Introduction:**

Excess body fatness is an established risk factor for various types of chronic disease and all-cause mortality. Most previous studies are based on body mass index (BMI) as a general measure of adiposity, but whether measures of central adiposity that better represent metabolically active visceral fat, such as waist-to-height ratio (WtHR), may be better at predicting disease and mortality risks is less known.

**Materials and Methods:**

Data from a large, prospective cohort in the U.S. including 50,618 women and 43,783 men (mean age of 67.3 years, predominantly non-Hispanic White), among whom 21,565 women and 26,758 men died during follow-up (1997–2018), were used to calculate multivariable-adjusted hazard rate ratios and 95% CIs for WtHR, BMI, and waist circumference in relation to total and cause-specific mortality.

**Results:**

WtHR was strongly correlated with BMI (r = 0.81). After adjustment for BMI and other covariates, WtHR (≥0.55 vs. < 0.50) was positively associated with all-cause mortality risk in women (RR = 1.23, 95% CI 1.17–1.29) and men (RR = 1.11, 95% CI 1.06–1.17). BMI and WC were also independently, positively associated with subsequent mortality risk at a similar magnitude to WtHR. Associations persisted for all grouped causes of death in women and men, with the exception of cancer and Alzheimer’s disease mortality in men. Mortality associations with WtHR were generally stronger among individuals younger than age 70 years compared to older individuals.

**Discussion:**

WtHR was associated with all-cause, cardiovascular disease, cancer, respiratory disease, and Alzheimer’s disease mortality in women and men at a magnitude similar to BMI or WC. Excess adiposity is an established major risk factor for premature death, but different measures may better predict mortality in different populations defined by age or other factors.

## Introduction

Excess body fatness is an established risk factor for premature mortality and for several chronic diseases including cardiovascular disease, type II diabetes, and at least 13 types of cancer [1–3]. Most studies have relied on body mass index (BMI, kg/m^2^) to estimate excess body fatness due to ease of measurement, ability to compare across different study populations, and its strong correlation to various aspects of excess body fatness (r > 0.7) [4]. However, whether other measures of excess body fatness that focus more on central adiposity, such as waist circumference (WC) or waist to height ratio (WtHR), may better measure visceral fat and therefore be more predictive of future disease risk is unclear [5].

Many previous studies of abdominal adiposity have focused on WC and most have shown that WC predicts mortality risk, with and without adjusting for BMI. In the largest study to date [6], mortality risk increased by 7% per 5 cm increase in waist circumference among men and by 9% per 5 cm increase among women. Given WC does not account for differences in height, more recent studies have begun examining WtHR as another potential measure of excess adiposity [7]. Though limited, studies to date have reported WtHR associations with mortality and cardiometabolic risk, often with better discriminatory power than BMI or WC [7–9]. Individual studies have been relatively small, but in a recent meta-analysis [10] including 20 studies, all-cause mortality increased by 16% when examining WtHR continuously (per 1 standard deviation). Furthermore, studies have suggested that keeping waist circumference to less than half of one’s height is associated with additional years of life [8] and leading the European Association for the Study of Obesity to recommend the use of WtHR > 0.5 as part of the diagnosis and treatment planning of obesity [11].

While WtHR has been gaining attention in the scientific community as a potentially better predictor of overall chronic disease risk compared to BMI or WC, the studies to date are relatively small and insufficient to draw conclusions, particularly for cause-specific mortality. Thus, we examined associations between WtHR, WC, and BMI in relation to all-cause mortality as well as grouped causes of mortality that are among the most common causes of death including cardiovascular disease, cancer, respiratory diseases, and Alzheimer’s disease/dementia in a large prospective cohort of US men and women.

## Materials and methods

Men and women in this analysis were drawn from the 184,190 participants in the CPS-II Nutrition Cohort, a prospective study of cancer incidence and mortality established by the American Cancer Society in 1992 as a subgroup of the larger 1982 CPS-II baseline mortality cohort [12]. Most participants were age 50–74 years at enrollment in 1992. At baseline, they completed a ten-page, self-administered, mailed questionnaire that included questions on demographic, medical, behavioral, environmental, and dietary factors. Beginning in 1997, follow-up questionnaires were sent to cohort members every two years to update exposure information and to ascertain newly diagnosed cancers. All follow-up questionnaire response rates (after multiple mailings) among living cohort members were at least 88%. End of mortality follow-up for the present analysis was December 31, 2018. All aspects of CPS-II have been reviewed and approved by the Emory University Institutional Review Board.

For this analysis, participants were eligible for inclusion if they returned the 1997 follow-up survey when waist circumference was measured (n = 160,300). We subsequently excluded participants with missing waist circumference or BMI (n = 26,813), underweight BMI < 18.5 kg/m^2^ (n = 1,222), top/bottom 0.1% of waist circumference or BMI (n = 397), missing smoking status (n = 1,714), and history of cancer or emphysema at study entry in 1997 (n = 35,753). Underweight was excluded because there were too few individuals in this group and their low BMI may be due to underlying illness. Top/bottom 0.1% of WC and BMI were excluded because these extremes were deemed likely implausible values. After exclusions, the final analytic cohort consisted of 50,618 women and 43,783 men with a mean age of 67.3 years (± 6.2 SD) at the start of this study in 1997.

The primary endpoint was death from any cause occurring between the 1997 survey return date and December 31, 2018. Deaths were identified through biennial automated linkage of the entire cohort with the National Death Index [13]. Death certificates or codes for cause of death have been obtained for 98.7% of all known deaths. Causes of death were classified by using the International Classification of Diseases (ICD), Tenth Revision [14]. Specific primary causes of death were grouped into 5 broad categories: cardiovascular disease (codes I00–I99); cancer (codes C00–C76 and C80–C97); respiratory diseases (codes J00-J999); Alzheimer’s disease and dementia (codes F00-F059, G30-G309); and all other causes combined. Grouped causes of death were examined if an adequate number of deaths were available for analysis. There were 48,323 deaths from all causes (21,565 in women, 26,758 in men) that occurred by the end of follow-up.

BMI (weight in kg/height in m^2^) at baseline in 1997 was calculated using self-reported weight and height and categorized as follows: 18.5- < 22.0, 22.0- < 25.0 (reference), 25.0- < 27.5, 27.5- < 30, and ≥ 30.0 kg/m^2^. Participants were sent a tape measure and asked to measure their waist circumference (just above the navel for women and at the navel for men) in inches (in.), round down to the nearest quarter of an inch, and record the measurement on their survey. Waist circumference was categorized as <30.25, 30.25- < 33.50, 33.50- < 37.00, and ≥ 37.00 inches for women and <36.00, 36.00- < 38.25, 38.25- < 41.25, and ≥41.25 inches for men. WtHR was calculated as waist circumference (in.) divided by height (in.) and categorized as <0.50 (reference), 0.50- < 0.55, and ≥0.55, consistent with the European Association for the Study of Obesity recommendation [11].

Multivariable adjusted Cox proportional hazards regression models were used to compute hazard ratios (HR) corresponding 95% confidence intervals (CI) for associations between various measures of excess body fatness (WtHR, BMI and WC) and mortality outcomes (all-cause, cardiovascular disease, cancer, respiratory diseases, Alzheimer’s disease/dementia, and all other causes combined) in men and women separately. Person-years of follow-up for each participant were computed as the time from the 1997 survey return date to date of death or end of follow-up on December 31, 2018, whichever came first. For each specific exposure variable, risk was assessed in 3 models: [1] adjusted for age only (single year) and [2] additionally adjusted for other potential confounders found to be relevant in previous CPS-II studies of obesity including: smoking (status and time since quitting among former smokers), physical activity MET-hours per week, diet score based on American Cancer Society dietary guidelines [15], alcohol intake, and pre-existing comorbidities score (includes type II diabetes, hypertension, high cholesterol, and cardiovascular disease; coded as 0, 1, 2+), and 3) additionally adjusted for BMI in WC and WtHR. Models among women were also adjusted for age at menopausal status, postmenopausal hormone use (PMH), and parity.

A stratified analysis by age (<70, ≥ 70 years) and a sensitivity analysis restricted to lifelong nonsmokers were also conducted. Finally, an analysis reexamining WtHR using sex-specific quartiles instead of the pre-defined cutpoints to allow for the differences in the distribution of WtHR values by sex. Statistical Analysis Software (SAS) Studio Version 9.4 was used for all analyses, and analyses were performed using de-identified data between March 1, 2023- November 1, 2024.

## Results

Among women, the mean BMI was 25.8 kg/m^2^ (SD ± 4.6), mean waist circumference was 34.1 in (SD ± 5.0) and mean WtHR was 0.53 (SD ± 0.08). Among men, the mean BMI was 26.5 kg/m^2^ (SD ± 3.7), mean waist circumference was 38.9 in (SD ± 3.9) and mean WtHR was 0.55 (SD ± 0.05). WtHR was strongly correlated with BMI (r = 0.81). Men and women with greater excess body fatness according to WtHR had a greater number of comorbidities, engaged in less moderate-to-vigorous physical activity, were less likely to consume any alcohol, and had a lower overall diet quality score (Table 1).

**Table 1:** Select baseline participant characteristics by categories of waist-to-height ratio among women and men in the Cancer Prevention Study-II Nutrition Cohort, 1997.

	Wasit to Height Ratio					
	Women			Men		
Characteristic	< 0.50 (N = 19,811)	0.50- < 0.55 (N = 12,971)	≥ 0.55 (N = 17,836)	< 0.50 (N = 5,925)	0.50- < 0.55 (N = 17,113)	≥0.55 (N = 20,745)
**Comorbidity Score, N (%)**						
0	8,373 (42.3)	3,823 (29.5)	3,774 (21.2)	2,237 (37.8)	4,990 (29.2)	4,638 (22.4)
1	7,672 (38.7)	5,306 (40.9)	6,695 (37.5)	2,071 (34.9)	6,001 (35.1)	7,029 (33.9)
2+	3,766 (19.0)	3,842 (29.6)	7,367 (41.3)	1,617 (27.3)	6,122 (35.8)	9,078 (43.8)
**BMI (kg/m** ^2^ **), N (%)**						
18.5 – < 22.0	9,444 (47.7)	1,030 (7.9)	174 (1.0)	2,247 (37.9)	989 (5.8)	88 (0.4)
22.0 – < 25.0	7,997 (40.4)	5,005 (38.6)	1,681 (9.4)	3,090 (52.1)	7,547 (44.1)	1,664 (8.0)
25.0 – < 27.5	2,017 (10.2)	4,748 (36.6)	4,103 (23.0)	550 (9.3)	6,725 (39.3)	6,295 (30.3)
27.5 – < 30	280 (1.4)	1,562 (12.0)	4,193 (23.5)	31 (0.5)	1,600 (9.3)	6,205 (29.9)
30+	73 (0.4)	626 (4.8)	7,685 (43.1)	7 (0.1)	252 (1.5)	6,493 (31.3)
**Physical Activity (Mets/Week), N (%)**						
None	760 (3.8)	685 (5.3)	1,629 (9.1)	282 (4.8)	923 (5.4)	1,949 (9.4)
0- < 7.5	6,300 (31.8)	4,963 (38.3)	7,805 (43.8)	1,457 (24.6)	4,981 (29.1)	7,708 (37.2)
7.5+	12,504 (63.1)	7,092 (54.78)	8,010 (44.9)	4,067 (68.6)	10,834 (63.3)	10,512 (50.7)
Unknown	247 (1.2)	231 (1.8)	392 (2.2)	119 (2.0)	375 (2.2)	576 (2.8)
**Smoking Status, N (%)**						
Never Smoker	11,146 (56.3)	7,358 (56.7)	10,354 (58.0)	2,540 (42.9)	6,231 (36.4)	6,455 (31.1)
Former Smoker, quit less than 20 years ago	2,674 (13.5)	1,975 (15.2)	3,008 (16.9)	789 (13.3)	3,043 (17.8)	4,824 (23.2)
Former Smoker, quit 20- < 30 years ago	1,833 (9.2)	1,119 (8.6)	1,530 (8.6)	704 (11.9)	2,419 (14.1)	3,483 (16.8)
Former Smoker, quit 30 + years ago	3,118 (15.7)	1,854 (14.3)	2,189 (12.3)	1,501 (25.3)	4,635 (27.1)	5,120 (24.7)
Current Smoker, < 40 years duration	375 (1.9)	213 (1.6)	239 (1.3)	76 (1.3)	158 (0.9)	168 (0.8)
Current Smoker, ≥ 40 years duration	665 (3.4)	452 (3.5)	516 (2.9)	315 (5.3)	627 (3.7)	695 (3.3)
**Alcohol Intake, N (%)**						
Not current or <1 drink per week	11,361 (57.3)	8,420 (64.9)	13,435 (75.3)	2,798 (47.2)	8,274 (48.3)	10,999 (53.0)
1-6 Drinks per week	6,219 (31.4)	3,270 (25.2)	3,131 (17.5)	1,930 (32.6)	5,315 (31.1)	5,537 (26.7)
1 + Drink per day	2,231 (11.3)	1,281 (9.9)	1,270 (7.1)	1,197 (20.2)	3,524 (20.6)	4,209 (20.3)
	**Waist to Height Ratio**					
	**Women**			**Men**		
	**< 0.50**	**0.50- < 0.55**	**≥ 0.55**	**< 0.50**	**0.50- < 0.55**	**≥0.55**
**ACS Diet Score, N (%)**						
0-2	3,580 (18.1)	2,949 (22.7)	5,021 (28.1)	950 (16.0)	3,153 (18.4)	5,046 (24.3)
3-5	8,921 (45.0)	5,906 (45.5)	7,931 (44.5)	2,350 (39.7)	7,460 (43.6)	9,200 (44.3)
6-9	5,993 (30.2)	3,215 (24.8)	3,418 (19.2)	2,171 (36.6)	5,182 (30.3)	4,712 (22.7)
Missing	1,317 (6.6)	901 (6.9)	1,466 (8.2)	454 (7.7)	1,318 (7.7)	1,787 (8.6)
**Age at Interview (1997), Mean (SD)**	65.75 (6.3)	66.76 (6.3)	66.96 (6.3)	67.86 (5.9)	68.35 (5.8)	68.43 (5.7)
**Waist Circumference (in), Mean (SD)**	29.56 (2.0)	33.73 (1.6)	39.35 (3.6)	33.96 (1.6)	36.92 (1.6)	41.84 (3.2)

After adjustment for BMI and other covariates, WtHR was positively associated with all-cause mortality risk in women (≥0.55 vs. < 0.50 RR = 1.23, 95% CI 1.17–1.29) and men (RR = 1.11, 95% CI 1.06–1.17) (Table 2 and 3, respectively). Multivariable-adjusted relative risks were positively associated with all grouped causes of death in women with similar magnitude of association for all causes of death examined (Table 2). In men, WtHR was positively associated with all causes of mortality except cancer and Alzheimer’s disease (Table 3). BMI and WC were also independently, positively associated with subsequent mortality risk at a similar magnitude to WtHR (S1–S2 Tables in S1 File). In women and men, BMI ≥ 30 kg/m^2^ was associated with 22% higher risk of total mortality compared to normal weight (BMI 22- < 25 kg/m^2^), and positive associations were seen for all grouped causes of death other than Alzheimer’s disease. WC was also positively associated with total mortality and cause-specific mortality other than Alzheimer’s disease in both women and men at a magnitude similar to that of BMI and WtHR.

**Table 2:** Multivariable adjusted hazard ratios (HR) and corresponding 95% confidence intervals (CI) for the association between waist to height ratio and mortality, women.

	Waist to Height Ratio		
	< 0.50	0.50 – < 0.55	≥0.55
**All Cause Death**			
Deaths/ Total person-years	7,354/104,509	5,382/75,711	8,829/121,189
Age-adjusted, HR (95% CI)	1.00 (Referent)	1.03 (1.00, 1.07)	1.31 (1.27, 1.35)
Multivariate adjusted^1^, HR (95% CI)	1.00 (Referent)	0.96 (0.93, 1.00)	1.12 (1.09, 1.16)
Multivariate adjusted plus BMI, HR (95% CI)	1.00 (Referent)	1.05 (1.01, 1.10)	1.23 (1.17, 1.29)
**Cancer**			
Deaths/ Total person-years	1,746/21,812	1,227/14,951	1,963/23,319
Age-adjusted, HR (95% CI)	1.00 (Referent)	1.05 (0.97, 1.12)	1.28 (1.20, 1.36)
Multivariate adjusted^1^, HR (95% CI)	1.00 (Referent)	1.02 (0.95, 1.10)	1.22 (1.14, 1.31)
Multivariate adjusted plus BMI, HR (95% CI)	1.00 (Referent)	1.04 (0.96, 1.14)	1.21 (1.10, 1.34)
**Cardiovascular Disease**			
Deaths/ Total person-years	2,363/33,983	1,864/26,420	3,171/44,123
Age-adjusted, HR (95% CI)	1.00 (Referent)	1.08 (1.02, 1.15)	1.42 (1.35, 1.50)
Multivariate adjusted^1^, HR (95% CI)	1.00 (Referent)	0.97 (0.92, 1.04)	1.14 (1.07, 1.20)
Multivariate adjusted plus BMI, HR (95% CI)	1.00 (Referent)	1.06 (0.99, 1.13)	1.21 (1.11, 1.31)
**Coronary Heart Disease**			
Deaths/Total person-years			
Age-adjusted, HR (95% CI)	783/10,764	682/9,170	1,226/16,239
Multivariate adjusted^1^, HR (95% CI)	1.00 (Referent)	1.19 (1.08, 1.32)	1.65 (1.51, 1.80)
Multivariate adjusted plus BMI, HR (95% CI)	1.00 (Referent)	1.04 (0.94, 1.16)	1.24 (1.12, 1.36)
**Stroke**			
Deaths/ Total person-years	1.00 (Referent)	1.13 (1.01, 1.27)	1.36 (1.19, 1.55)
Age-adjusted, HR (95% CI)	1.00 (Referent)	1.03 (0.91, 1.16)	1.09 (0.98, 1.21)
Multivariate adjusted^1^, HR (95% CI)	1.00 (Referent)	0.95 (0.85, 1.07)	0.93 (0.83, 1.04)
Multivariate adjusted plus BMI, HR (95% CI)	1.00 (Referent)	1.09 (0.95, 1.25)	1.17 (0.99, 1.37)
**Respiratory Disease**			
Deaths/ Total person-years	594/8,773	415/6,171	697/9,910
Age-adjusted, HR (95% CI)	1.00 (Referent)	0.98 (0.86, 1.11)	1.28 (1.14, 1.43)
Multivariate adjusted^1^, HR (95% CI)	1.00 (Referent)	0.90 (0.79, 1.02)	1.08 (0.96, 1.21)
Multivariate adjusted plus BMI, HR (95% CI)	1.00 (Referent)	1.03 (0.89, 1.19)	1.18 (1.00, 1.40)
**Alzheimer’s Disease and Dementia**			
Deaths/Total person-years	1,283/20,455	825/13,217	1,093/17,738
Age-adjusted, HR (95% CI)	1.00 (Referent)	0.88 (0.81, 0.96)	0.91 (0.84, 0.99)
Multivariate adjusted^1^, HR (95% CI)	1.00 (Referent)	0.86 (0.79, 0.94)	0.87 (0.79, 0.94)
Multivariate adjusted plus BMI, HR (95% CI)	1.00 (Referent)	1.02 (0.92, 1.13)	1.18 (1.04, 1.33)
**All Other Causes Combined**			
Deaths/ Total person-years	1,368/19,486	1,051/14,952	1,905/26,099
Age-adjusted, HR (95% CI)	1.00 (Referent)	1.09 (1.01, 1.19)	1.53 (1.43, 1.64)
Multivariate adjusted^1^, HR (95% CI)	1.00 (Referent)	1.00 (0.92, 1.08)	1.25 (1.16, 1.34)
Multivariate adjusted plus BMI, HR (95% CI)	1.00 (Referent)	1.11 (1.01, 1.22)	1.36 (1.22, 1.51)

^1^Multivariate adjusted w/o BMI is adjusted for age, parity, PMH use and kind, ACS diet score, alcohol intake, smoking status, physical activity, comorbidity score, and age at menopause.

**Table 3:** Multivariable adjusted hazard ratios (HR) and corresponding 95% confidence intervals (CI) for the association between waist to height ratio and mortality, men.

	Waist to Height Ratio		
	< 0.50	0.50 – < 0.55	≥0.55
**All Cause Death**			
Deaths/ Total person-years	3,305/41,318	9,953/126,692	13,500/169,107
Age-adjusted, HR (95% CI)	1.00 (Referent)	1.00 (0.96, 1.04)	1.21 (1.16, 1.25)
Multivariate adjusted^1^, HR (95% CI)	1.00 (Referent)	0.94 (0.90, 0.98)	1.04 (1.00, 1.08)
Multivariate adjusted plus BMI, HR (95% CI)	1.00 (Referent)	1.02 (0.98, 1.07)	1.11 (1.05, 1.17)
**Cancer**			
Deaths/ Total person-years	802/9,580	2,426/28,852	3,114/36,220
Age-adjusted, HR (95% CI)	1.00 (Referent)	1.02 (0.94, 1.10)	1.15 (1.06, 1.24)
Multivariate adjusted^1^, HR (95% CI)	1.00 (Referent)	0.98 (0.91, 1.06)	1.05 (0.97, 1.14)
Multivariate adjusted plus BMI, HR (95% CI)	1.00 (Referent)	0.95 (0.87, 1.04)	0.94 (0.85, 1.04)
**Cardiovascular Disease**			
Deaths/ Total person-years	1,140/13,776	3,651/45,369	5,335/65,072
Age-adjusted, HR (95% CI)	1.00 (Referent)	1.06 (0.99, 1.13)	1.38 (1.29, 1.47)
Multivariate adjusted^1^, HR (95% CI)	1.00 (Referent)	0.96 (0.90, 1.03)	1.13 (1.05, 1.20)
Multivariate adjusted plus BMI, HR (95% CI)	1.00 (Referent)	1.04 (0.97, 1.13)	1.17 (1.07, 1.28)
**Coronary Heart Disease**			
Deaths/ Total person-years	566/6,210	1,829/21,520	2,818/32,583
Age-adjusted, HR (95% CI)	1.00 (Referent)	1.06 (0.97, 1.17)	1.45 (1.32, 1.59)
Multivariate adjusted^1^, HR (95% CI)	1.00 (Referent)	0.95 (0.86, 1.04)	1.14 (1.04, 1.25)
Multivariate adjusted plus BMI, HR (95% CI)	1.00 (Referent)	1.08 (0.97, 1.20)	1.29 (1.14, 1.46)
**Stroke**			
Deaths/ Total person-years	188/2,385	613/7,572	776/9,415
Age-adjusted, HR (95% CI)	1.00 (Referent)	1.08 (0.91, 1.27)	1.22 (1.04, 1.43)
Multivariate adjusted^1^, HR (95% CI)	1.00 (Referent)	1.03 (0.87, 1.21)	1.08 (0.92, 1.27)
Multivariate adjusted plus BMI, HR (95% CI)	1.00 (Referent)	1.10 (0.91, 1.32)	1.17 (0.95, 1.45)
**Respiratory Disease**			
Deaths/ Total person-years	302/4,007	812/10,922	1,111/14,822
Age-adjusted, HR (95% CI)	1.00 (Referent)	0.89 (0.78, 1.01)	1.08 (0.95, 1.23)
Multivariate adjusted^1^, HR (95% CI)	1.00 (Referent)	0.83 (0.73, 0.95)	0.90 (0.79, 1.03)
Multivariate adjusted plus BMI, HR (95% CI)	1.00 (Referent)	1.01 (0.87, 1.18)	1.15 (0.96, 1.37)
**Alzheimer’s Disease and Dementia**			
Deaths/ Total person-years	376/5,457	995/14,853	1,064/16,299
Age-adjusted, HR (95% CI)	1.00 (Referent)	0.87 (0.77, 0.98)	0.85 (0.75, 0.96)
Multivariate adjusted^1^, HR (95% CI)	1.00 (Referent)	0.85 (0.75, 0.96)	0.80 (0.71, 0.90)
Multivariate adjusted plus BMI, HR (95% CI)	1.00 (Referent)	1.03 (0.90, 1.18)	1.08 (0.92, 1.27)
**All Other Causes Combined**			
Deaths/ Total person-years	685/8,498	2,069/26,696	2,876/36,695
Age-adjusted, HR (95% CI)	1.00 (Referent)	1.01 (0.92, 1.10)	1.25 (1.15, 1.36)
Multivariate adjusted^1^, HR (95% CI)	1.00 (Referent)	0.95 (0.87, 1.03)	1.07 (0.98, 1.16)
Multivariate adjusted plus BMI, HR (95% CI)	1.00 (Referent)	1.08 (0.98, 1.19)	1.21 (1.08, 1.36)

^1^Multivariate adjusted without BMI is adjusted for age, ACS diet score, alcohol intake, smoking status, physical activity, and comorbidity score.

In both women and men, associations between WtHR and overall and cause-specific mortality outcomes were largely unchanged when restricted to lifelong nonsmokers whereas associations with BMI and WC became stronger for cancer and respiratory disease mortality among nonsmokers (Table 4). In analyses stratified by age (<70, ≥ 70 years), associations for all mortality outcomes were modestly stronger for younger women compared to older women (all-cause <70 years RR = 1.20, 95% CI 1.20–1.39 vs. ≥ 70 years RR = 1.17, 95% CI 1.07–1.23, p-interaction <0.01) (S3 Table). On the contrary, the magnitude of associations with WtHR and mortality appeared slightly stronger among older men (<70 years RR = 1.03, 95% CI 0.95–1.12 vs. ≥ 70 years RR = 1.15, 95% CI 1.07–1.23, p-interaction = 0.38), and associations for cardiovascular disease and respiratory disease mortality among younger men were no longer statistically significant (S4 Table). S5–6 Tables show associations between sex-specific quartiles of WtHR and mortality risks. When using sex-specific quartiles, associations between the lowest and highest quartiles are more pronounced than when using pre-defined cutpoints, particularly among women. Finally, to address the potential for reverse causation, we examined associations excluding the first two years of follow-up and tested for an interaction with follow-up time, and results remained unchanged (data not shown).

**Table 4:** Multivariable adjusted hazard ratios (HR) and corresponding 95% confidence intervals (CI) for the association between waist to height ratio and mortality among non-smokers for women and men.

	Waist to Height Ratio for Life-Long Non-Smokers					
	Women			Men		
	< 0.50	0.50 – 0.55	≥0.55	< 0.50	0.50 – 0.55	≥0.55
**All Cause Death**						
Number of Deaths	4,007	2,969	4,956	1,234	3,235	3,893
Multivariate Adjusted, Model, HR (95% CI)	1.00 (Referent)	1.07 (1.01, 1.13)	1.24 (1.16, 1.32)	1.00 (Referent)	1.05 (0.97, 1.13)	1.14 (1.04, 1.24)
**Cancer Death**						
Number of Deaths	823	591	958	255	653	760
Multivariate Adjusted Model, HR (95% CI)	1.00 (Referent)	1.09 (0.96, 1.23)	1.20 (1.04, 1.39)	1.00 (Referent)	0.94 (0.80, 1.11)	0.95 (0.78, 1.16)
**CVD Death**						
Number of Deaths	1,385	1,095	1,831	454	1,257	1,599
Multivariate Adjusted, Model, HR (95% CI)	1.00 (Referent)	1.05 (0.96, 1.15)	1.19 (1.07, 1.32)	1.00 (Referent)	1.09 (0.96, 1.23)	1.18 (1.02, 1.36)
** CHD Death**						
Number of Deaths	466	382	684	214	601	801
Multivariate Adjusted, Model, HR (95% CI)	1.00 (Referent)	1.05 (0.90, 1.22)	1.27 (1.07, 1.52)	1.00 (Referent)	1.18 (0.99, 1.40)	1.35 (1.10, 1.66)
Model, HR (95% CI)						
** Stroke Death**						
Number of Deaths	375	309	408	81	229	243
Multivariate Adjusted, Model, HR (95% CI)	1.00 (Referent)	1.16 (0.98, 1.38)	1.18 (0.96, 1.46)	1.00 (Referent)	1.07 (0.80, 1.43)	1.03 (0.73, 1.45)
**Respiratory Death**						
Number of Deaths	259	192	354	71	211	258
Multivariate Adjusted, Model, HR (95% CI)	1.00 (Referent)	1.07 (0.86, 1.33)	1.31 (1.02, 1.67)	1.00 (Referent)	1.23 (0.91, 1.67)	1.29 (0.90, 1.85)
**Alzheimer’s Disease and Dementia Death**						
Number of Deaths	759	499	681	165	352	357
Multivariate Adjusted, Model, HR (95% CI)	1.00 (Referent)	1.05 (0.92, 1.19)	1.20 (1.03, 1.40)	1.00 (Referent)	0.98 (0.79, 1.22)	1.08 (0.83, 1.40)
**All Other Cause Death**						
Number of Deaths	781	592	1,132	289	762	919
Multivariate Adjusted, Model, HR (95% CI)	1.00 (Referent)	1.10 (0.98, 1.25)	1.37 (1.19, 1.58)	1.00 (Referent)	1.07 (0.92, 1.25)	1.24 (1.03, 1.49)

*Women’s models are adjusted for age, parity, PMH use and kind, ACS diet score, alcohol intake, physical activity, comorbidity score, age at menopause, and BMI.

*Men’s models are adjusted for age, ACS diet score, alcohol intake, physical activity, comorbidity score, and BMI.

### Discussion

In this study, the largest to date, WtHR was associated with all-cause, cardiovascular disease, cancer, respiratory disease, and Alzheimer’s disease mortality in women and all causes except cancer and Alzheimer’s disease mortality in men at a magnitude similar to BMI or WC. Associations remained largely unchanged after adjustment for BMI further supporting that central adiposity is independently associated with mortality risk. These findings are largely consistent with some, but not all, previous studies.

In a previous study including 21,109 participants using the National Health and Nutrition Examination Survey (NHANES), WtHR was associated with incidence of hypertension, dyslipidemia, and diabetes, but was only associated with cardiovascular disease, cancer, and all-cause mortality among women aged ≥ 65 years [16]. In contrast, in a British study including 7,414 women and men, WtHR was a better predictor of subsequent mortality and a greater number of years of life lost than BMI [8]. In that study, authors concluded “Keep your waist circumference to less than half your height.” A recent meta-analysis including 17 studies that included 74,520 total participants found that WtHR similarly predicted hypertension, dyslipidemia, and hyperglycemia compared to BMI and WC [17]. Another recent meta-analysis reported 16% higher mortality risk per one standard deviation of WtHR (95% CI 1.07–1.25), a risk estimate similar to that reported in the present study [10]. Importantly, not all of the causes of death examined in this study have been previously studied, thus additional studies are needed to confirm these results. Of note, the lack of association with Alzheimer’s disease mortality in men requires further investigation as no previous study has reported on this outcome. Additionally, while the lack of association with cancer mortality in men was also observed in the previous NHANES study [16], it could be due to the contribution of individual cancers to the whole (e.g., a large number of lung cancers would likely attenuate overall cancer associations).

WtHR was more strongly associated with risk among women under age 70 years. While there was no statistically significant difference by age among men, associations appeared to be stronger in men aged 70 years or older. These results are similar to a prior study of BMI and mortality risk in this same study population [18], where we found that BMI was most strongly associated with mortality before age 70 years. Disease- and age-related weight loss, which tends to be driven by loss of lean mass, may make BMI a poorer measure of excess mortality risk in older adults [19], whereas abdominal adiposity, even as overall weight decreases, is retained as adults age. Thus, WC and WtHR may be better predictors of disease risk in older adults. This is similarly seen in individuals who smoke cigarettes, because they tend to be leaner overall but retain visceral fat [20]. In the present study, associations with WtHR and mortality did not substantively change when restricting to lifelong nonsmokers, suggesting that smoking history is not confounding the association whereas in our previous study of BMI, associations were strongest among lifelong non-smokers [18] While it is unclear why WtHR may not be confounded by smoking history and studies are needed to elucidate the underlying mechanisms, these findings suggest that different measures of adiposity may be more appropriate to use depending on underlying patient characteristics.

There are several strengths that should be noted. This was the largest study to date examining WtHR, and collection of multiple measures of adiposity allowed for direct comparison of these measures in the same population. Other strengths include the ability to account for various confounders and long-term follow-up. The study has notable limitations. Specifically, the main limitation is the reliance on a single self-reported measure of weight and height. However, when compared with measured weight and height, self-reported data tended to overestimate height in men and underestimate weight in women, but the magnitude of this error is likely small [21]. Additionally, waist circumference was self-measured by participants, but past studies have found that self-recorded waist circumference data are valid and reliable [22].

In conclusion, in the largest study to date to examine WtHR in relation to mortality, WtHR, WC, and BMI similarly predicted all-cause, cardiovascular disease, cancer, respiratory disease, and Alzheimer’s disease mortality. While excess adiposity has been established as a major risk factor for premature death, different measures of excess adiposity that can all be easily assessed in clinical settings may be more predictive in different populations defined by age or other factors. Additional studies are needed to understand differences by sex, age, race and ethnicity, as well as other factors such as smoking history.

## Supporting information

S1 File Supplementary Table 1. Multivariable adjusted hazard ratios (HR) and corresponding 95% confidence intervals (CI) for the association between body mass index (BMI) and mortality and waist circumference and mortality, women. **Supplementary Table 2**. Multivariable adjusted hazard ratios (HR) and corresponding 95% confidence intervals (CI) for the association between body mass index (BMI) and mortality and waist circumference and mortality for men.
(DOCX)
