# Supplementary material for: Is waist to height ratio better at assessing cause-specific mortality risk than body mass index or waist circumference? A prospective analysis in a large U.S.-based cohort
Source: PLoS One. 2025 Aug 13;20(8):e0328760. doi: 10.1371/journal.pone.0328760 (PMC12349718; doi:10.1371/journal.pone.0328760)
Supplement: S1 File — Multivariable adjusted hazard ratios (HR) and corresponding 95% confidence intervals (CI) for the association between body mass index (BMI) and mortality and waist circumference and mortality, women. Supplementary Table 2. Multivariable adjusted hazard ratios (HR) and corresponding 95% confidence intervals (CI) for the association between body mass index (BMI) and mortality and waist circumference and mortality for men. (DOCX) [file pone.0328760.s001.docx]

**Supplemental Table 1**: Multivariable adjusted hazard ratios (HR) and corresponding 95% confidence intervals (CI) for the association between body mass index (BMI) and mortality and waist circumference and mortality, women.

|  | **BMI (kg/m^2^)** | | | | | **Waist Circumference (in)** | | | |
| --- | --- | --- | --- | --- | --- | --- | --- | --- | --- |
|  | **18.5 - <22** | **22 -< 25** | **25 - <27.5** | **27.5 - < 30** | **30+** | **< 30.25** | **30.25-33.50** | **33.50 – 37.00** | **≥37.00** |
| **All Cause Deaths** | 1.14 (1.10, 1.18) | 1.00 (Referent) | 0.99 (0.95, 1.03) | 1.05 (1.00, 1.10) | 1.23 (1.18, 1.28) | 1.00 (Referent) | 1.08 (1.04, 1.13) | 1.22 (1.16, 1.28) | 1.42 (1.35, 1.51) |
| **Cancer Deaths** | 1.05 (0.97, 1.14) | 1.00 (Referent) | 1.06 (0.98, 1.15) | 1.16 (1.05, 1.28) | 1.27 (1.16, 1.39) | 1.00 (Referent) | 1.08 (0.99, 1.19) | 1.19 (1.07, 1.31) | 1.28 (1.13, 1.44) |
| **CVD Deaths** | 1.12 (1.05, 1.20) | 1.00 (Referent) | 1.00 (0.93, 1.07) | 1.02 (0.95, 1.11) | 1.28 (1.19, 1.37) | 1.00 (Referent) | 1.06 (0.99, 1.15) | 1.20 (1.10, 1.31) | 1.44 (1.31, 1.58) |
| **CHD Deaths** | 1.02 (0.91, 1.14) | 1.00 (Referent) | 0.97 (0.87, 1.08) | 0.99 (0.87, 1.13) | 1.24 (1.11, 1.40) | 1.00 (Referent) | 1.06 (0.93, 1.21) | 1.24 (1.07, 1.43) | 1.44 (1.23, 1.69) |
| **Stroke Deaths** | 1.28 (1.12, 1.45) | 1.00 (Referent) | 1.03 (0.90, 1.17) | 0.93 (0.79, 1.10) | 0.95 (0.81, 1.11) | 1.00 (Referent) | 1.25 (1.08, 1.45) | 1.39 (1.17, 1.64) | 1.56 (1.29, 1.89) |
| **Respiratory Deaths** | 1.23 (1.08, 1.41) | 1.00 (Referent) | 0.90 (0.78, 1.04) | 1.04 (0.88, 1.23) | 1.34 (1.16, 1.56) | 1.00 (Referent) | 1.19 (1.02, 1.39) | 1.22 (1.02, 1.46) | 1.59 (1.30, 1.95) |
| **Alzheimer’s Disease and Dementia Death** | 1.23 (1.13, 1.35) | 1.00 (Referent) | 0.89 (0.81, 0.99) | 0.88 (0.77, 1.00) | 0.81 (0.72, 0.92) | 1.00 (Referent) | 1.04 (0.93, 1.16) | 1.17 (1.03, 1.32) | 1.29 (1.12, 1.49) |
| **All Other Cause Death** | 1.16 (1.07, 1.27) | 1.00 (Referent) | 1.02 (0.93, 1.11) | 1.12 (1.01, 1.25) | 1.38 (1.25, 1.51) | 1.00 (Referent) | 1.11 (1.00, 1.23) | 1.34 (1.20, 1.50) | 1.64 (1.44, 1.86) |

*BMI models are adjusted for age, parity, PMH use and kind, ACS diet score, alcohol intake, smoking status, physical activity, comorbidity score and age at menopause.

*WC models are adjusted for age, parity, PMH use and kind, ACS diet score, alcohol intake, smoking status, physical activity, comorbidity score, age at menopause, and BMI.

**Supplemental Table 2**: Multivariable adjusted hazard ratios (HR) and corresponding 95% confidence intervals (CI) for the association between body mass index (BMI) and mortality and waist circumference and mortality for men.

|  | **BMI (kg/m^2^)** | | | | | **Waist Circumference (in)** | | | |
| --- | --- | --- | --- | --- | --- | --- | --- | --- | --- |
|  | **18.5 - <22** | **22 - < 25** | **25 - < 27.5** | **27.5 - < 30** | **30+** | **< 36.00** | **36.00-38.25** | **38.25-41.25** | **≥41.25** |
| **All Cause Deaths** | 1.17 (1.12, 1.23) | 1.00 (Referent) | 0.97 (0.94, 1.00) | 1.04 (1.00, 1.08) | 1.22 (1.17, 1.26) | 1.00 (Referent) | 1.13 (1.09, 1.18) | 1.20 (1.15, 1.25) | 1.38 (1.31, 1.45) |
| **Cancer Deaths** | 0.96 (0.87, 1.07) | 1.00 (Referent) | 0.99 (0.93, 1.06) | 1.09 (1.01, 1.18) | 1.18 (1.09, 1.28) | 1.00 (Referent) | 1.06 (0.98, 1.15) | 1.10 (1.01, 1.20) | 1.15 (1.04, 1.28) |
| **CVD Deaths** | 1.15 (1.07, 1.25) | 1.00 (Referent) | 1.00 (0.95, 1.05) | 1.11 (1.05, 1.18) | 1.32 (1.24, 1.40) | 1.00 (Referent) | 1.13 (1.06, 1.20) | 1.19 (1.11, 1.28) | 1.44 (1.32, 1.56) |
| **CHD Deaths** | 1.22 (1.09, 1.36) | 1.00 (Referent) | 0.97 (0.91, 1.05) | 1.11 (1.03, 1.21) | 1.28 (1.17, 1.39) | 1.00 (Referent) | 1.11 (1.01, 1.22) | 1.18 (1.07, 1.30) | 1.44 (1.28, 1.61) |
| **Stroke Deaths** | 1.06 (0.87, 1.28) | 1.00 (Referent) | 0.96 (0.85, 1.10) | 1.00 (0.86, 1.17) | 1.09 (0.93, 1.29) | 1.00 (Referent) | 1.31 (1.12, 1.54) | 1.27 (1.06, 1.52) | 1.41 (1.14, 1.74) |
| **Respiratory Deaths** | 1.44 (1.23, 1.67) | 1.00 (Referent) | 0.93 (0.83, 1.04) | 0.96 (0.84, 1.10) | 1.13 (0.98, 1.30) | 1.00 (Referent) | 1.18 (1.03, 1.35) | 1.30 (1.12, 1.51) | 1.58 (1.33, 1.89) |
| **Alzheimer’s Disease and Dementia Death** | 1.33 (1.16, 1.52) | 1.00 (Referent) | 0.84 (0.76, 0.93) | 0.83 (0.74, 0.94) | 0.84 (0.73, 0.98) | 1.00 (Referent) | 1.17 (1.04, 1.32) | 1.10 (0.96, 1.26) | 1.21 (1.02, 1.43) |
| **All Other Cause Death** | 1.24 (1.12, 1.37) | 1.00 (Referent) | 0.97 (0.90, 1.04) | 0.98 (0.90, 1.07) | 1.29 (1.19, 1.41) | 1.00 (Referent) | 1.19 (1.09, 1.30) | 1.34 (1.22, 1.47) | 1.56 (1.39, 1.74) |

*BMI models are adjusted for age, ACS diet score, alcohol intake, smoking status, physical activity, and comorbidity score.

*Waist circumference models are adjusted for age, ACS diet score, alcohol intake, smoking status, physical activity, comorbidity score, and BMI.

**Supplemental Table 3:** Multivariable adjusted hazard ratios (HR) and corresponding 95% confidence intervals (CI) for the association between waist to height ratio and mortality among women <70 years old and among women ≥ 70 years old.

|  | **< 70 years old (N = 33,944)** | | | **≥ 70 years old (N = 16,674)** | | |
| --- | --- | --- | --- | --- | --- | --- |
|  | **< 0.50** | **0.50 – 0.55** | **≥ 0.55** | **< 0.50** | **0.50 – 0.55** | **≥0.55** |
| **All Cause Death**  **#** of Deaths  Multivariate Adjusted Model,  HR (95% CI) | 3,444  1.00  (Referent) | 2,263  1.04 (0.98, 1.11) | 3,967  1.29 (1.20, 1.39) | 3,910  1.00 (Referent) | 3,119  1.05 (0.99, 1.10) | 4,862  1.17 (1.10, 1.25) |
| **Cancer Death**  **#** of Deaths  Multivariate Adjusted Model,  HR (95% CI) | 1,099  1.00 (Referent) | 684  1.01 (0.90, 1.13) | 1,149  1.26 (1.10, 1.44) | 647  1.00 (Referent) | 543  1.08 (0.95, 1.23) | 814  1.14 (0.98, 1.33) |
| **CVD Death**  **#** of Deaths  Multivariate Adjusted Model,  HR (95% CI) | 890  1.00 (Referent) | 638  1.05 (0.93, 1.19) | 1,215  1.33 (1.16, 1.52) | 1,473  1.00 (Referent) | 1,226  1.04 (0.96, 1.14) | 1,956  1.13 (1.03, 1.25) |
| **CHD Death**  **#** of Deaths  Multivariate Adjusted Model,  HR (95% CI) | 282  1.00 (Referent) | 216  1.07 (0.87, 1.31) | 462  1.51 (1.20, 1.90) | 501  1.00 (Referent) | 466  1.14 (0.99, 1.32) | 764  1.27 (1.08, 1.50) |
| **Stroke Death**  **#** of Deaths  Multivariate Adjusted Model,  HR (95% CI) | 266  1.00 (Referent) | 190  1.18 (0.95, 1.48) | 245  1.22 (0.93, 1.60) | 389  1.00 (Referent) | 302  1.04 (0.87, 1.23) | 427  1.13 (0.93, 1.39) |
| **Respiratory Death**  **#** of Deaths  Multivariate Adjusted Model,  HR (95% CI) | 275  1.00 (Referent) | 182  1.00 (0.80, 1.25) | 307  1.01 (0.77, 1.33) | 319  1.00 (Referent) | 233  1.01 (0.84, 1.23) | 390  1.29 (1.03, 1.61) |
| **Alzheimer’s Disease and Dementia Death**  **#** of Deaths  Multivariate Adjusted Model,  HR (95% CI) | 518  1.00  (Referent) | 289  1.02 (0.86, 1.21) | 406  1.24 (1.01, 1.52) | 765  1.00 (Referent) | 536  1.00 (0.89, 1.14) | 687  1.12 (0.97, 1.31) |
| **All Other Cause Death**  **#** of Deaths  Multivariate Adjusted Model,  HR (95% CI) | 662  1.00  (Referent) | 470  1.13 (0.99, 1.31) | 890  1.44 (1.22, 1.70) | 706  1.00 (Referent) | 581  1.08 (0.95, 1.22) | 1,015  1.29 (1.11, 1.49) |

* Models are adjusted for age, parity, PMH use and kind, ACS diet score, alcohol intake, physical activity, comorbidity score, age at menopause, and BMI.

**Supplemental Table 4:** Multivariable adjusted hazard ratios (HR) and corresponding 95% confidence intervals (CI) for the association between waist to height ratio and mortality among men <70 years old and among men ≥ 70 years old.

|  | **< 70 years old (N = 25,536)** | | | **≥ 70 years old (N = 18,247)** | | |
| --- | --- | --- | --- | --- | --- | --- |
|  | **< 0.50** | **0.50 – 0.55** | **≥ 0.55** | **< 0.50** | **0.50 – 0.55** | **≥0.55** |
| **All Cause Death**  **#** of Deaths  Multivariate Adjusted Model,  HR (95% CI) | 1,457  1.00 (Referent) | 4,070  0.95 (0.89, 1.02) | 5,901  1.03 (0.95, 1.12) | 1,848  1.00 (Referent) | 5,883  1.07 (1.01, 1.13) | 7,599  1.15, (1.07, 1.23) |
| **Cancer Death**  **#** of Deaths  Multivariate Adjusted Model,  HR (95% CI) | 441  1.00 (Referent) | 1,264  0.91 (0.80, 1.03) | 1,649  0.89 (0.77, 1.03) | 361  1.00 (Referent) | 1,162  1.00 (0.87, 1.14) | 1,465  1.00 (0.85, 1.16) |
| **CVD Death**  **#** of Deaths  Multivariate Adjusted Model,  HR (95% CI) | 436  1.00 (Referent) | 1,270  0.96 (0.84, 1.08) | 2,113  1.10 (0.95, 1.27) | 704  1.00 (Referent) | 2,381  1.08 (0.98, 1.19) | 3,222  1.19 (1.06, 1.32) |
| **CHD Death**  **#** of Deaths  Multivariate Adjusted Model,  HR (95% CI) | 206  1.00 (Referent) | 643  1.04 (0.86, 1.24) | 1,117  1.26 (1.03, 1.54) | 360  1.00 (Referent) | 1,186  1.08 (0.95, 1.24) | 1,701  1.28 (1.10, 1.49) |
| **Stroke Death**  **#** of Deaths  Multivariate Adjusted Model,  HR (95% CI) | 82  1.00 (Referent) | 219  1.01 (0.75, 1.36) | 311  1.12 (0.79, 1.58) | 106  1.00 (Referent) | 394  1.16 (0.91, 1.47) | 465  1.19 (0.90, 1.57) |
| **Respiratory Death**  **#** of Deaths  Multivariate Adjusted Model,  HR (95% CI) | 110  1.00 (Referent) | 286  1.01 (0.78, 1.31) | 462  1.27 (0.94, 1.71) | 192  1.00 (Referent) | 526  0.99 (0.82, 1.20) | 649  1.05 (0.84, 1.32) |
| **Alzheimer’s Disease and Dementia Death**  **#** of Deaths  Multivariate Adjusted Model,  HR (95% CI) | 146  1.00 (Referent) | 346  0.93 (0.75, 1.17) | 368  0.92 (0.70, 1.21) | 230  1.00 (Referent) | 649  1.08 (0.91, 1.29) | 696  1.18 (0.96, 1.45) |
| **All Other Cause Death**  **#** of Deaths  Multivariate Adjusted Model,  HR (95% CI) | 324  1.00 (Referent) | 904  1.01 (0.87, 1.17) | 1,309  1.12 (0.95, 1.33) | 361  1.00 (Referent) | 1,165  1.15 (1.00, 1.31) | 1,567  1.29 (1.10, 1.50) |

* Models adjusted for ACS diet score, alcohol intake, physical activity, comorbidity score, and BMI

|  | **Waist to Height Ratio** | | | |
| --- | --- | --- | --- | --- |
|  | **≤0.47** | **>0.47-0.52** | **>0.52-0.58** | **≥0.58** |
| **All Cause Death** |  |  |  |  |
| Death / Total Person years | 4,600 / 65,473 | 5,127 / 72,812 | 5,743 / 79,842 | 6,095 / 83,281 |
| Age-adjusted, HR (95% CI) | 1.00 (Referent) | 1.03 (0.99, 1.07) | 1.12 (1.08, 1.17) | 1.39 (1.34, 1.45) |
| Multivariate adjusted w/o BMI, HR (95% CI) | 1.00 (Referent) | 0.97 (0.93, 1.01) | 1.01 (0.97, 1.05) | 1.16 (1.11, 1.20) |
| Multivariate adjusted, HR (95% CI) | 1.00 (Referent) | 1.07 (1.02, 1.12) | 1.16 (1.11, 1.22) | 1.31 (1.23, 1.39) |
| **Cancer Death** |  |  |  |  |
| Death / Total Person years | 1,096 / 13,695 | 1,205 / 14,997 | 1,304 / 15,483 | 1,331 / 15,907 |
| Age-adjusted, HR (95% CI) | 1.00 (Referent) | 1.07 (0.99, 1.17) | 1.16 (1.07, 1.25) | 1.34 (1.24, 1.45) |
| Multivariate adjusted w/o BMI, HR (95% CI) | 1.00 (Referent) | 1.05 (0.97, 1.14) | 1.12 (1.03, 1.21) | 1.27 (1.17, 1.38) |
| Multivariate adjusted, HR (95% CI) | 1.00 (Referent) | 1.09 (1.00, 1.20) | 1.16 (1.04, 1.29) | 1.26 (1.11, 1.42) |
| **Cardiovascular Disease Death** |  |  |  |  |
| Death / Total Person years | 1,458 / 21,008 | 1,720 / 24,726 | 1,988 / 27,935 | 2,232 / 30,858 |
| Age-adjusted, HR (95% CI) | 1.00 (Referent) | 1.06 (0.99, 1.14) | 1.18 (1.10, 1.26) | 1.56 (1.46, 1.67) |
| Multivariate adjusted w/o BMI, HR (95% CI) | 1.00 (Referent) | 0.96 (0.90, 1.03) | 1.00 (0.93, 1.07) | 1.18 (1.10, 1.26) |
| Multivariate adjusted, HR (95% CI) | 1.00 (Referent) | 1.05 (0.97, 1.13) | 1.14 (1.04, 1.24) | 1.30 (1.17, 1.44) |
| **Coronary Heart Disease Death** |  |  |  |  |
| Death / Total Person years | 479 / 6,560 | 603 / 8,253 | 760 / 10,191 | 849 / 11,168 |
| Age-adjusted, HR (95% CI) | 1.00 (Referent) | 1.12 (0.99, 1.26) | 1.36 (1.22, 1.53) | 1.78 (1.59, 1.99) |
| Multivariate adjusted w/o BMI, HR (95% CI) | 1.00 (Referent) | 0.99 (0.88, 1.12) | 1.10 (0.98, 1.23) | 1.24 (1.10, 1.40) |
| Multivariate adjusted, HR (95% CI) | 1.00 (Referent) | 1.06 (0.93, 1.21) | 1.23 (1.07, 1.43) | 1.36 (1.15, 1.61) |
| **Stroke Death** |  |  |  |  |
| Death / Total Person years | 388 / 5,573 | 479 / 6,948 | 508 / 7,129 | 444 / 6,024 |
| Age-adjusted, HR (95% CI) | 1.00 (Referent) | 1.11 (0.97, 1.27) | 1.13 (0.99, 1.29) | 1.17 (1.02, 1.34) |
| Multivariate adjusted w/o BMI, HR (95% CI) | 1.00 (Referent) | 1.04 (0.91, 1.19) | 1.00 (0.88, 1.15) | 0.96 (0.83, 1.11) |
| Multivariate adjusted, HR (95% CI) | 1.00 (Referent) | 1.22 (1.05, 1.42) | 1.31 (1.11, 1.56) | 1.36 (1.11, 1.67) |
| **Respiratory Death** |  |  |  |  |
| Death / Total Person years | 352 / 5,217 | 430 / 6,397 | 427 / 6,193 | 497 / 7,047 |
| Age-adjusted, HR (95% CI) | 1.00 (Referent) | 1.12 (0.98, 1.29) | 1.09 (0.94, 1.25) | 1.48 (1.29, 1.70) |
| Multivariate adjusted w/o BMI, HR (95% CI) | 1.00 (Referent) | 1.04 (0.90, 1.20) | 0.95 (0.82, 1.10) | 1.20 (1.04, 1.39) |
| Multivariate adjusted, HR (95% CI) | 1.00 (Referent) | 1.22 (1.04, 1.42) | 1.22 (1.02, 1.46) | 1.44 (1.17, 1.79) |
| **Alzheimer’s Disease and Dementia Death** |  |  |  |  |
| Death / Total Person years | 831 / 13,226 | 826 / 13,256 | 826 / 13,276 | 718 / 11,652 |
| Age-adjusted, HR (95% CI) | 1.00 (Referent) | 0.90 (0.81, 0.99) | 0.86 (0.78, 0.95) | 0.90 (0.81, 0.99) |
| Multivariate adjusted w/o BMI, HR (95% CI) | 1.00 (Referent) | 0.88 (0.80, 0.97) | 0.83 (0.75, 0.91) | 0.84 (0.75, 0.93) |
| Multivariate adjusted, HR (95% CI) | 1.00 (Referent) | 1.01 (0.91, 1.13) | 1.08 (0.96, 1.22) | 1.26 (1.08, 1.46) |
| **All Other Death** |  |  |  |  |
| Death / Total Person years | 863 / 12,327 | 946 / 13,437 | 1,198 / 16,956 | 1,317 / 17,817 |
| Age-adjusted, HR (95% CI) | 1.00 (Ref) | 1.02 (0.93, 1.12) | 1.27 (1.16, 1.38) | 1.62 (1.49, 1.77) |
| Multivariate adjusted w/o BMI, HR (95% CI) | 1.00 (Ref) | 0.94 (0.86, 1.04) | 1.09 (0.99, 1.19) | 1.26 (1.15, 1.38) |
| Multivariate adjusted, HR (95% CI) | 1.00 (Ref) | 1.06 (0.96, 1.17) | 1.28 (1.14, 1.44) | 1.40 (1.23, 1.61) |

**Supplemental Table 5:** Multivariable adjusted hazard ratios (HR) and corresponding 95% confidence intervals (CI) for the association between sex-specific quartiles of WtHR and mortality, women.

^1^Multivariate adjusted w/o BMI is adjusted for age, parity, PMH use and kind, ACS diet score, alcohol intake, smoking status, physical activity, comorbidity score, and age at menopause. **Supplemental Table 6:** Multivariable adjusted hazard ratios (HR) and corresponding 95% confidence intervals (CI) for the association between sex-specific quartiles of WtHR and mortality, men.

|  | **Waist to Height Ratio** | | | |
| --- | --- | --- | --- | --- |
|  | **≤0.51** | **>0.51-0.55** | **>0.55-0.58** | **≥0.58** |
| **All Cause Death** |  |  |  |  |
| Death / Total Person years | 5,322 / 67,164 | 8,055 / 102,356 | 5,308 / 67,512 | 8,073 / 100,085 |
| Age-adjusted, HR (95% CI) | 1.00 (Referent) | 0.99 (0.96, 1.03) | 1.09 (1.05, 1.13) | 1.30 (1.25, 1.34) |
| Multivariate adjusted w/o BMI, HR (95% CI) | 1.00 (Referent) | 0.94 (0.91, 0.98) | 0.99 (0.95, 1.03) | 1.11 (1.07, 1.15) |
| Multivariate adjusted, HR (95% CI) | 1.00 (Referent) | 1.01 (0.97, 1.05) | 1.07 (1.02, 1.12) | 1.14 (1.09, 1.20) |
| **Cancer Death** |  |  |  |  |
| Death / Total Person years | 1,333 / 15,951 | 1,918 / 22,742 | 1,245 / 14,462 | 1,846 / 21,497 |
| Age-adjusted, HR (95% CI) | 1.00 (Referent) | 0.97 (0.90, 1.04) | 1.04 (0.96, 1.12) | 1.18 (1.10, 1.26) |
| Multivariate adjusted w/o BMI, HR (95% CI) | 1.00 (Referent) | 0.94 (0.87, 1.00) | 0.97 (0.90, 1.05) | 1.07 (0.99, 1.15) |
| Multivariate adjusted, HR (95% CI) | 1.00 (Referent) | 0.90 (0.83, 0.98) | 0.91 (0.82, 0.99) | 0.93 (0.84, 1.03) |
| **Cardiovascular Disease Death** |  |  |  |  |
| Death / Total Person years | 1,855 / 22,697 | 2,987 / 37,088 | 2,062 / 25,780 | 3,222 / 38,652 |
| Age-adjusted, HR (95% CI) | 1.00 (Referent) | 1.05 (0.99, 1.11) | 1.20 (1.13, 1.28) | 1.48 (1.40, 1.57) |
| Multivariate adjusted w/o BMI, HR (95% CI) | 1.00 (Referent) | 0.97 (0.92, 1.03) | 1.06 (0.99, 1.13) | 1.20 (1.13, 1.28) |
| Multivariate adjusted, HR (95% CI) | 1.00 (Referent) | 1.04 (0.97, 1.11) | 1.13 (1.04, 1.22) | 1.21 (1.11, 1.31) |
| **Coronary Heart Disease** |  |  |  |  |
| Death / Total Person years | 958 / 10,846 | 1,465 / 17,221 | 1,091 / 12,737 | 1,699 / 19,510 |
| Age-adjusted, HR (95% CI) | 1.00 (Referent) | 1.00 (0.92, 1.08) | 1.23 (1.13, 1.34) | 1.49 (1.38, 1.62) |
| Multivariate adjusted w/o BMI, HR (95% CI) | 1.00 (Referent) | 0.91 (0.84, 0.98) | 1.05 (0.96, 1.14) | 1.16 (1.07, 1.26) |
| Multivariate adjusted, HR (95% CI) | 1.00 (Referent) | 0.99 (0.90, 1.08) | 1.16 (1.05, 1.29) | 1.23 (1.10, 1.39) |
| **Stroke Death** |  |  |  |  |
| Death / Total Person years | 282 / 3,661 | 526 / 6,366 | 324 / 4,086 | 445 / 5,259 |
| Age-adjusted, HR (95% CI) | 1.00 (Referent) | 1.22 (1.05, 1.41) | 1.25 (1.06, 1.46) | 1.35 (1.17, 1.57) |
| Multivariate adjusted w/o BMI, HR (95% CI) | 1.00 (Referent) | 1.17 (1.01, 1.35) | 1.16 (0.98, 1.36) | 1.20 (1.03, 1.40) |
| Multivariate adjusted, HR (95% CI) | 1.00 (Referent) | 1.28 (1.09, 1.51) | 1.31 (1.08, 1.59) | 1.35 (1.09, 1.67) |
| **Respiratory Death** |  |  |  |  |
| Death / Total Person years | 456 / 6,076 | 670 / 8,997 | 427 / 5,667 | 672 / 9,012 |
| Age-adjusted, HR (95% CI) | 1.00 (Referent) | 0.96 (0.85, 1.08) | 1.01 (0.89, 1.16) | 1.26 (1.12, 1.42) |
| Multivariate adjusted w/o BMI, HR (95% CI) | 1.00 (Referent) | 0.91 (0.80, 1.02) | 0.90 (0.79, 1.03) | 1.03 (0.91, 1.17) |
| Multivariate adjusted, HR (95% CI) | 1.00 (Referent) | 1.08 (0.94, 1.24) | 1.14 (0.97, 1.34) | 1.31 (1.10, 1.57) |
| **Alzheimer’s Disease and Dementia Death** |  |  |  |  |
| Death / Total Person years | 589 / 8,611 | 791 / 11,853 | 466 / 7,198 | 589 / 8,947 |
| Age-adjusted, HR (95% CI) | 1.00 (Referent) | 0.87 (0.78, 0.97) | 0.86 (0.76, 0.97) | 0.88 (0.78, 0.98) |
| Multivariate adjusted w/o BMI, HR (95% CI) | 1.00 (Referent) | 0.85 (0.76, 0.94) | 0.83 (0.73, 0.93) | 0.82 (0.73, 0.92) |
| Multivariate adjusted, HR (95% CI) | 1.00 (Referent) | 1.00 (0.89, 1.13) | 1.05 (0.91, 1.22) | 1.10 (0.93, 1.30) |
| **All Other Death** |  |  |  |  |
| Death / Total Person years | 1,089 / 13,830 | 1,689 / 21,677 | 1,108 / 14,406 | 1,744 / 21,976 |
| Age-adjusted, HR (95% CI) | 1.00 (Referent) | 1.02 (0.95, 1.11) | 1.12 (1.03, 1.22) | 1.38 (1.27, 1.48) |
| Multivariate adjusted w/o BMI, HR (95% CI) | 1.00 (Referent) | 0.97 (0.90, 1.05) | 1.02 (0.93, 1.11) | 1.16 (1.08, 1.26) |
| Multivariate adjusted, HR (95% CI) | 1.00 (Referent) | 1.09 (1.00, 1.19) | 1.18 (1.06, 1.30) | 1.28 (1.15, 1.43) |

^1^Multivariate adjusted without BMI is adjusted for age, ACS diet score, alcohol intake, smoking status, physical activity, and comorbidity score.
